# Assessment of Behavioural Markers of Autonoetic Consciousness during Episodic Autobiographical Memory Retrieval: A Preliminary Analysis

**DOI:** 10.1155/2008/691925

**Published:** 2008-04-11

**Authors:** M. Irish, B. A. Lawlor, S. M. O'Mara, R. F. Coen

**Affiliations:** ^1^Trinity College Institute of NeuroscienceTrinity CollegeDublin 2Ireland; ^2^Mercer’s Institute for Research on AgeingSt. James's HospitalDublin 8Ireland

**Keywords:** Episodic memory, autobiographical memory, autonoetic consciousness, memory assessment

## Abstract

There is ongoing theoretical debate regarding episodic memory and how it can be accurately measured, in particular if the focus should be content-based recall of episodic details or something more experiential involving the subjective capacity to mentally travel back in time and “re-live” aspects of the original event. The autonoetic subscale of the Episodic Autobiographical Memory Interview (EAMI) is presented here as a new test instrument that attempts to redress theoretical and methodological shortcomings in autobiographical memory assessment. The EAMI merges a phenomenological detail-based approach with an assessment of autonoetic consciousness, departing considerably from traditional Remember/Know paradigms used within this field. We present findings from an initial pilot study investigating the potential markers of autonoetic consciousness that may accompany episodic retrieval. Key behavioural indices of autonoetic consciousness, notably those of viewer perspective, visual imagery, and emotional re-experiencing, emerged as being inextricably bound with the level of phenomenological detail recalled and the overall re-living judgment. The autonoetic subscale of the EAMI permits conceptually refined assessment of episodic personal memories and the accompanying subjective experience of mental re-living, characteristic of episodic memory.

## 1. Introduction

Autobiographical memory (ABM) is a complex construct comprising both episodic and semantic components, involving sensory-perceptual event details usually in the form of visual imagery [1]. The close personal relevance of such recalled events typically implies some evocation of the previously experienced emotion during the original occurrence [5]. Thus the recollection of certain past events may culminate in a rich sensory and emotional re-experiencing of the original event.

Tulving originally suggested that episodic recollection was inextricably bound with the accompanying recollective experience and could not be defined by content alone [8,9]. The hallmark of episodic retrieval was conceptualised as that of mentally travelling back in time to the original occurrence, and "re-living" the remembered event [10]. This is potentially mediated by a special type of awareness called autonoetic consciousness, which is facilitated by the frontal lobes and enables subjective mental time travel. Therefore, when we remember a personal memory from our past, the episodic memory system enables us to re-experience or re-live elements of, or all of the original occurrence, as

ISSN 0953-4180/08/$17.00 © 2008 – IOS Press and the authors. All rights reserved opposed to semantic recall of the event, divested of this contextually-rich autonoetic experience.

Where assessment of ABM is concerned, personal incidents are typically scored using a content-based approach according to three main criteria, namely "what", "where" and "when" (e.g. Kopelman et al. [3]) with the presence of all three elements considered to represent retrieval from episodic memory. However, no attempt is made to investigate the subjective mental experience accompanying the memory. Inclusion of the Remember/Know paradigm for these three elements of what/where/when, in an effort to measure the subjective phenomenal experience (e.g. Piolino et al. [7]), ultimately falls short as it reverts back to the content-based approach to validate the subjective mental experience itself. Dissociating the hypothetical constructs of event details and the subjective mental experience, and analysing them independently represents both a true conceptual and practical advance in understanding episodic memory.

The autonoetic subscale of the Episodic Autobiographical Memory Interview (EAMI), attempts to remedy the aforementioned conceptual and methodological shortcomings in ABM assessment, and to tease apart the potential behavioural markers of episodic ABMs. In this manner, the EAMI continues to grade ABMs based on the overall level of contextual detail, yet departs from conventional assessments that subscribe to the view that it is the detail and the detail alone that makes memories truly episodic [4].

## 2. Methods

### 2.1. Participants

30 healthy young control individuals aged 18–60 years (mean age: 25.63 years, 13 males:17 females) took part voluntarily in this study. A Chi-squared distribution goodness of fit test showed that males and females were represented equally throughout this sample ($\chi^2$(df: 1,29) $= 0.533$, $p = 0.465$).

### 2.2. Materials and procedure

The Episodic Autobiographical Memory Interview (EAMI) is a semi-structured interview, incorporating non-restrictive categories across the lifespan from Childhood (0–15 years), to Early Adulthood (16–30 years), to Middle Adulthood (31–45 years), to Later Adulthood (46 up to 5 years ago) to Recent memory (in the last 5 years). Personal Semantic information (Names of people, Daily Living, An Important Date) and recall of 3 Autobiographical Events per epoch are probed as dissociable elements (publication in preparation).

Table 1
Behavioural markers probed using autonoetic subscale of the EAMI

| Autonoetic marker | Variable coding |
|---|---|
| Viewer perspective | Own eyes |
| | Third person |
| | Both perspectives |
| Level of movement | Movement |
| | No movement |
| Vividness Scale | Very vivid to very vague |
| Emotional connection | Connected |
| | Detached |
| Reliving judgment | Reliving |
| | Partly reliving |
| | Everything but reliving |
| | Not reliving |

Here we focus on the piloting of an assessment of autonoetic consciousness to complement the recall of Autobiographical events. Participants were asked to recall in detail, one event that occurred during their Childhood Period (0–15 years) according to the following instructions adapted from Piolino et al. [6,7]:

> *"I would like you to describe out loud for me, with as much detail as possible, a particular event that happened to you during this time, something that stands out for you that you can remember well."*

If participants could not spontaneously recall a particular event, the following topics were suggested:

1. Event linked to school life
2. A family event
3. A religious occasion

Modified versions of Moscovitch et al.'s [4] "Event Details Checklist", and Johnson et al.'s [2] "Memory Characteristics Questionnaire" were used to probe the memories recalled using several discrete categories of detail. Potential autonoetic behavioural markers commonly associated with subjective re-living of memories were identified in the literature to form the autonoetic subscale, which required participants to comment on the visual representation of the memory and their accompanying emotional and subjective autonoetic experience if any (see Table 1). The test session lasted for approximately 8–10 minutes.

## 3. Results

Non-parametric statistical analyses (Pearson Chi-Square) were chosen as the variables under considera-

Table 2
Breakdown by gender of level of detail, emotional connection and re-experiencing judgments

| | Level of detail recalled (%) | | Emotional connection (%) | | Re-experiencing judgments (%) | |
|---|---|---|---|---|---|---|
| | High | Low | Connected | Detached | Reliving | Not Reliving |
| Male | 37 | 100 | 18.7 | 71.4 | 20 | 66.7 |
| Female | 63 | 0 | 81.3 | 28.6 | 80 | 33.3 |
| Significance Level | $P < 0.05$ | | $P < 0.005$ | | $P < 0.01$ | |

tion were largely categorical in nature. The data were analysed using SPSS Edition 14.0.

### 3.1. *Level of detail and subjective recollective experience*

A score of 1 point was awarded for the provision of each discrete detail in accordance with the scoring system of Moscovitch et al.'s [4] "Event Details Checklist". The detail scores assigned to each memory were collapsed into categories of "low" and "high" levels of detail, comprising scores of 0–4 and 5–7 points respectively. Low detail memories thus reflected the recall of core details such as what, where, when, and the implication of event, whereas high detail memories indicated the additional recall of phenomenological elements such as sensory, emotional, and metacognitive details. The relationship between the level of detail and accompanying recollective experience was significant, $\chi^2$ (df: 3, 29) $= 7.778$, $p = 0.05$, with 100% of low detail memories not re-lived by participants.

### 3.2. *Level of detail and viewer perspective of visual representation*

A robust relationship was found, with 100% of "low-detail" memories viewed from a third person perspective, and 74.1% of "high-level" detail memories viewed from "own eyes" perspective, $\chi^2$ (df: 2, 29) $= 9.167$, $p = 0.01$.

### 3.3. *Recollective experience and viewer perspective of visual representation*

A relationship between reliving judgments and the perspective taken by participants during recall of the event was evident with 86.7% of re-lived memories being viewed from "own eyes" perspective, $\chi^2$ (df: 2, 29) $= 6.300$, $p = 0.043$.

### 3.4. *Emotional connection and subjective recollective experience*

There is a highly significant relationship between re-living judgments and accompanying emotional connection during recall, $\chi^2$ (df: 3, 29) $= 26.652$, $p = 0.000$, with 100% of re-living judgments accompanied by emotional connection and 93.3% of not re-living judgments reported as "emotionally detached".

### 3.5. *Emotional connection and viewer perspective of visual representation*

There was a significant relationship between emotional connection and the accompanying viewer perspective in the visual representation of the memory, $\chi^2$ (df: 2, 29) $= 7.600$, $p = 0.022$, with 87.5% of emotionally connected memories viewed through "own eyes" perspective.

### 3.6. *Effect of gender*

There was a significant effect of gender, $\chi^2$ (df: 1, 29) $= 8.438$, $p = 0.004$, with a greater percentage of females reporting emotional connection during recall than males. Females tended to recall memories with a higher level of contextual detail, $\chi^2$ (df: 1, 29) $=$ 4.359, $p = 0.037$, and endorsed re-living the memories more so than males, $\chi^2$ (df: 1, 29) $= 6.652$, $p = 0.010$ (see Table 2).

### 3.7. *Level of movement and the subjective recollective experience*

There was no significant predictive effect of movement in the visual representation on the reliving judgments given by participants, $\chi^2$ (df: 1, 29) $= 1.677$, $p = 0.195$. This may reflect the fact that the majority of memories recalled by participants in this study incorporated movement in the visual representation (76.7%).

## 4. Discussion

The autonoetic subscale of the Episodic Autobiographical Memory Interview (EAMI) permits assessment of a number of key behavioural indices considered indicative of when a person mentally re-lives a past event via frontally-mediated autonoetic consciousness. In the current study, these markers emerged as being related to the visual imagery of the recalled memory and the viewer perspective taken during recall. Furthermore, emotional re-connection to the original event, with some element of the original emotion being re-experienced by participants during recall, was predictive of re-living judgments. Moreover, there was a significant gender effect, whereby females were more likely to re-experience emotions of the original event, recall more contextually-detailed memories and endorse a re-living judgment during recollection.

The current findings provide robust behavioural data to complement much of the theorising in the literature regarding episodic memory recall and the autonoetic subjective experience. We have demonstrated that whilst contextual details are an important feature of episodic memory, we must depart from the conventional content-based approach by emphasising the importance of the subjective mental experience and emotional re-experiencing during episodic recollection, consistent with Tulving's original thesis that episodic memory cannot be defined by content alone [8,9].

Episodic memory should be taken to indicate not only the capacity to represent a specific event and locate it in time and space, but also the capacity to re-live some specific feature of that event. We agree with current assertions that episodic memory is defined by the nature of the conscious awareness that accompanies retrieval, infusing the memory with emotions, thoughts and the subjectivity of the person remembering [11]. Therefore, current methods of assessment overly relying on content fall short. Our autonoetic assessment has been developed to provide an ecologically valid method of assessing personal episodic memory with emphasis on both content and the accompanying recollective experience, thus distinguishing this instrument from currently available measures.
